# Melioidosis in the Western Indian Ocean and the Importance of Improving Diagnosis, Surveillance, and Molecular Typing

**DOI:** 10.3390/tropicalmed3010030

**Published:** 2018-03-07

**Authors:** Andriniaina Rakotondrasoa, Mohammad Iqbal Issack, Benoît Garin, Fabrice Biot, Eric Valade, Pierre Wattiau, Nicolas Allou, Olivier Belmonte, Jastin Bibi, Erin P. Price, Jean-Marc Collard

**Affiliations:** 1 Unité de Bactériologie Expérimentale, Institut Pasteur de Madagascar, Antananarivo 101, Madagascar; aina@pasteur.mg; 2 Central Health Laboratory, Victoria Hospital, Candos 72211, Mauritius; moissack@yahoo.com; 3 Laboratoire Immuno-Hématologie, CHU Pointe-à-Pitre/Abymes 97159, Guadeloupe; benoitgarin@gmail.com; 4 Unité de Bactériologie/UMR_MD1, Institut de Recherche Biomédicale des Armées, Brétigny sur Orge, Ecole du Val-de-Grâce, 91223 Paris, France; fbiot.irba@defense.gouv.fr (F.B.); eric.valade@defense.gouv.fr (E.V.); 5 Unit of Foodborne, Highly Pathogenic Bacterial Zoonoses & Antibiotic Resistance, Veterinary and Agrochemical Research Center, Brussels 1180, Belgium; Pierre.Wattiau@coda-cerva.be; 6 Réanimation polyvalente, Centre Hospitalier Universitaire Félix Guyon, 97499 Saint Denis, France; nicolas.allou@hotmail.fr; 7 Bactériologie, Centre Hospitalier Universitaire Félix Guyon, 97499 Saint Denis, France; olivier.belmonte@chu-reunion.fr; 8 Ministry of Health, Public Health Department, Victoria, Seychelles; jastin.bibi@health.gov.sc; 9 Faculty of Science, Health, Education and Engineering, University of the Sunshine Coast, Sippy Downs, QLD 4556, Australia; eprice@usc.edu.au

**Keywords:** Melioidosis, *Burkholderia pseudomallei*, western Indian Ocean, diagnosis, MLST, Madagascar, Mauritius, Réunion, Seychelles

## Abstract

Melioidosis, caused by the bacterium *Burkholderia pseudomallei*, is an infectious disease of humans or animals, and the specific environmental conditions that are present in western Indian Ocean islands are particularly suitable for the establishment/survival of *B. pseudomallei*. Indeed, an increasing number of new cases have been reported in this region (Madagascar, Mauritius, Réunion (France), and Seychelles, except Comoros and Mayotte (France)), and are described in this review. Our review clearly points out that further studies are needed in order to investigate the real incidence and burden of melioidosis in the western Indian Ocean and especially Madagascar, since it is likely to be higher than currently reported. Thus, research and surveillance priorities were recommended (i) to improve awareness of melioidosis in the population and among clinicians; (ii) to improve diagnostics, in order to provide rapid and effective treatment; (iii) to implement a surveillance and reporting system in the western Indian Ocean; and (iv) to investigate the presence of *B. pseudomallei* in environmental samples, since we have demonstrated its presence in soil samples originating from the yard of a Madagascan case.

## 1. Introduction and History of Melioidosis in the Western Indian Ocean 

Melioidosis (also known as Whitmore’s disease or Nightcliff gardener’s disease) is an infectious disease that can infect humans or animals. This disease is caused by the bacterium *Burkholderia pseudomallei*. A recent study mapped documented human and animal cases and the presence of environmental *B. pseudomallei* in the world, in order to estimate the global burden of melioidosis by a formal modeling framework and assess the environmental suitability for *B. pseudomallei* [1]. Human cases are mainly reported in hyperendemic areas of Southeast Asia and northern Australia, with sporadic reports in parts of the Americas, Africa, Pacific Ocean islands, southern China, Hong Kong and Taiwan, and South Asia. It has been predicted that the specific environmental conditions which are present in western Indian Ocean islands are particularly suitable for the establishment/survival of *B. pseudomallei* [1], and an increasing number of new cases have been reported in this region [2]. A map of the western Indian Ocean with its different islands can be accessed at the website [3]. It was long ago hypothesized that the trade of (healthy or sick) animal carriers could contribute to the spread of the disease to non-endemic areas.

In Thailand, melioidosis is considered a disease predominantly of rice farmers, which is brought about by their frequent exposure to environments containing *B. pseudomallei*. The organism is a saprophyte found in soil and water, and people become infected, particularly after heavy rains, by contact with contaminated soil or water through percutaneous inoculation, by inhalation, or even through ingestion of contaminated water (e.g., aspiration in near-drowning events during flooding or tsunamis, or consumption of unchlorinated water). Person-to-person transmission can occur, albeit very rarely, through contact with blood and body fluids of an infected person. Infected people generally have underlying predisposing conditions, such as diabetes mellitus, renal disease, cirrhosis, thalassemia, cystic fibrosis, chronic obstructive lung disease, or immunosuppressive therapy. Clinical presentations vary widely, and include skin and soft tissue abscesses, pneumonia, and disseminated infection with septic shock, the latter having mortality rates above 80% [4]. The diverse clinical manifestations and the inadequacy of conventional bacterial identification methods render *B. pseudomallei* identification difficult, particularly in regions where this organism is not well-recognized, and could result in a serious underestimation of the disease in low-income countries, where capacity building is needed in the health services, as recently discussed by Limmathurotsakul et al. [1].

In Madagascar, the first isolation of *B. pseudomallei* occurred in Antananarivo in 1932 when Girard, a former Director of the Pasteur Institute of Madagascar, examined a dead guineapig inoculated with a submaxillary node of a slaughtered pig [5]. 

The presence of this bacterium in Madagascar was also reported later (1977) by Galimand and Dodin working in the ‘Whitmore bacillus laboratory’ in the Pasteur Institute in Paris. They isolated and identified the bacterium from soil samples originating from the zoo of Antananarivo and from a pig farm in the same city [6]. There are two isolates (770429-id6- and Soil1977-id7-) in the *B. pseudomallei* multilocus sequencing typing (MLST) database (https://pubmlst.org/bpseudomallei/) originating from soil collected from the Antananarivo region in 1977. These isolates correspond to ST-6 (alleles: 1, 1, 7, 2, 4, 5, 1) and ST-27 (alleles: 1, 3, 19, 4, 5, 1, 1), respectively [7]. ST-6 has not been reported in any other isolate to date; however, ST-27 was also found in a 1978 soil sample from Chantilly, France, during a devastating and highly unusual melioidosis outbreak (the so-called ‘Affaire du Jardin des Plantes’) that lasted several years and that resulted in the death or slaughter of dozens of animals across several zoos and equestrian clubs, probably by the transport of infected animals and/or contaminated manure [8]. A case of animal melioidosis, also related to the French outbreak, occurred in 1979 in a horse that had recently been imported from France into Réunion Island [6]. This epizootic of melioidosis across France and beyond was extraordinary, and greatly extended the known boundaries of this disease, being the first report of this disease in a temperate region [6].

Subsequently, between 2004 and 2017, there were six human cases of melioidosis that were detected and probably acquired in the city of Mahajanga (Madagascar), of which three were diagnosed and treated in Réunion Island in 2004, 2005, and 2016. Considering other islands of the western Indian Ocean, there were two cases in Mauritius in 2004 and 2006, one autochthonous case in 2012, and four imported cases in Réunion Island (three from Madagascar, in 2004, 2005, and 2016 respectively, and one from South Asia in 2017), and finally, two cases in cooks from the same facility in Seychelles in 2013. These cases are described in more detail in the next section. Since the cases were all investigated separately, some methodologies used for culturing and identifying *B. pseudomallei* were slightly different between the different laboratories concerned. We also describe the isolation of *B. pseudomallei* in the yard of case #9 from Madagascar.

## 2. Review of the Human Cases

N.B. Cases are numbered chronologically, independently of the country of origin.

### 2.1. Madagascar

Case #1. The first human case of melioidosis in the region was detected in May 2004 in a 60-year-old Frenchman living in Mahajanga, a city on the northwestern coast of Madagascar, along the Mozambique Channel, who had been transferred to a hospital in Réunion Island. He was admitted to an intensive care unit for management of respiratory distress and septic shock. About 20 or 30 years before the onset of the disease, he had lived for an unknown period of time in Vietnam, where melioidosis is endemic. He was a heavy drinker and smoker with cachexia. Four blood cultures and one bronchoalveolar lavage specimen, taken the first day after admission, were positive for *B. pseudomallei* [9]. The initial treatment was a combination of piperacillin and ciprofloxacin (the initial identification was ‘*Pseudomonas* sp.’), followed by ceftazidime when *B. pseudomallei* was recognized. Interestingly, two secondary cases (cases #2 and #3), acquired 150 and 180 days after the initial case and transmitted by a fiberoptic bronchoscope, were detected. A manufacturing defect rendered the decontamination processes of the bronchoscope insufficiently effective, and culture with subsequent DNA restriction analysis confirmed the secondary transmissions. These nosocomial transmissions concerned an 81-year-old man suffering from a chronic pulmonary disease, and a woman undergoing glucocorticoid therapy for lupus. Both secondary cases were also treated with ceftazidime (120 mg/kg/day) until recovery, followed by trimethoprim/sulfamethoxazole for 20 weeks.

Case #5. The second case in Madagascar was a 58-year-old Frenchman with an unremarkable medical history who had lived for the previous five years in Antananarivo [10]. The patient, a retired manager, had spent most of his life in France, and he had traveled for short periods in Tunisia, Turkey, and Mauritius (six years previously). His first symptoms (high fever with cough) appeared during a two-week holiday trip to Mahajanga in March 2005. He was initially admitted to a hospital in Antananarivo, where he was treated for ten days with amoxicillin/clavulanic acid and levofloxacin after a chest X-ray showed a patchy infiltrate of the right upper and middle lobes. His sputum acid-fast stain was negative. After clinical improvement, the patient was discharged, but five days later he became feverish again, and was referred to a hospital in Réunion Island for further management. Although four blood cultures were negative, *B. pseudomallei* was isolated from his bronchoalveolar lavage. He responded to treatment, which firstly comprised imipenem for two weeks, followed by oral trimethoprim/sulfamethoxazole and doxycycline. He was discharged at day 20 on eradication treatment with trimethoprim/sulfamethoxazole for a further five months. 

Cases #8 and #9. The third and fourth cases in Madagascar were rural farmers aged 52 and 45 years, respectively [11]. They were both admitted to the University hospital in Mahajanga with sepsis in July 2012 and in May 2013, respectively. One of them had an opacity in the lower left lung and a pleural effusion, whilst the other developed hepatic failure after one week of admission. They were both suffering from diabetes mellitus, and hepatomegaly and splenomegaly were detected by abdominal ultrasound examination during their hospitalization. The diagnosis of *B. pseudomallei* was possible thanks to a specific research study on melioidosis that was being conducted in this hospital during the period 2012–2013, which supported the purchase of consumables, such as blood culture bottles and culture media (chocolate, blood and Ashdown agars). However, both patients died despite antibiotic treatment.

Case #10. The fifth case in Madagascar was a 44-year-old Frenchman admitted with acute anuria and fever to the emergency service of a Belgian hospital near the French border in March 2013 [12]. He had a severe inflammatory syndrome, spondylodiscitis at L1, lung infiltrates, pyelonephritis, and a prostatic abscess. He was treated with a fluoroquinolone after the isolation of an organism initially identified as *B. cepacia* on a VITEK II instrument (bioMérieux, La Balme-Les-Grottes, France) from his blood cultures. The patient’s clinical condition remained stable, with decreasing inflammatory syndrome and fever, and he was discharged for few days until the real causative agent was identified. When *B. pseudomallei* was correctly identified by MALDI-TOF MS using the security-relevant reference library, and subsequently confirmed by a highly-specific PCR assay, the patient was readmitted to the hospital, the antimicrobial treatment was immediately changed (to meropenem 1 g iv t.i.d. and trimethoprim/sulfamethoxazole 2 × 160/800 mg t.i.d.) and his condition improved. Interestingly, the patient, a gardener by profession, reported frequent travels to his secondary residence in Mahajanga, usually for 3 weeks, every 3 or 4 months. 

Case #13. The last case from Madagascar was recently described by Allou et al. [2], and concerned a 63-year-old Frenchman, with no significant past medical history, who lived in Madagascar and made frequent trips to Mayotte, an island between Madagascar and Mozambique. After his admission to Mahajanga Hospital, where he was treated with ofloxacin for cough and fever lasting one week, his condition worsened (vomiting, dehydration, deterioration of consciousness) and he was transferred to an ICU at Saint Denis Hospital, Réunion Island, with septic shock and coma on admission. Briefly, a total body-computed tomography (TB-CT) scan showed multiple bilateral lung abscesses, a liver abscess, splenomegaly, and hepatomegaly. Despite immediate antimicrobial treatment (meropenem, colistin, amikacin), the evolution was marked by multiple organ failure and the patient died on day one. Blood cultures and respiratory samples were positive for a *Burkholderia* identified as *B. thailandensis* by a MALDI-TOF mass spectrometer (Bruker Biotyper, Bruker Daltonics, Bremen, Germany) but confirmed later as *B. pseudomallei* by qPCR targeting three genetic markers of the type III secretion system (*orf1*, *orf13*, and *BsSCU2*). It is noteworthy that *B. pseudomallei* was not at that time included in the IVD MALDI Biotyper database but in a separate database (SR: security-relevant) which was not available at the hospital. The strain was susceptible in vitro to ticarcillin/clavulanic acid, trimethoprim/sulfamethoxazole, and ceftazidime, and was resistant to meropenem and levofloxacin. The meropenem resistance (MIC = 12 mL/L) was unusual, and the presence of a possible metallo β-lactamase (MBL) was investigated with the RUO Etest MBL MP/MPI US 8/2 (bioMérieux, La Balme-Les-Grottes, France), but failed. The higher MIC values to different antibiotics suggested that this resistance could be due to a non-specific mechanism, such as efflux or impermeability.

The six cases are summarized in Table 1. After investigations, all cases were likely to have been acquired in Mahajanga, a favorite tourist destination in Madagascar located on the country’s northwestern coast on the Mozambique Channel (15°43′ S, 46°18′ E), 550 km from Antananarivo. Typing of strains isolated from five human cases by multilocus sequencing typing (MLST; Godoy et al. [7]) showed new sequence types (STs), all of which were possibly related to each other (varying by 1, 2, or 3 alleles, depending on the combinations).

### 2.2. Soil Investigation in Mahajanga, Madagascar, in the Yard of Case #10

**Figure A1:**
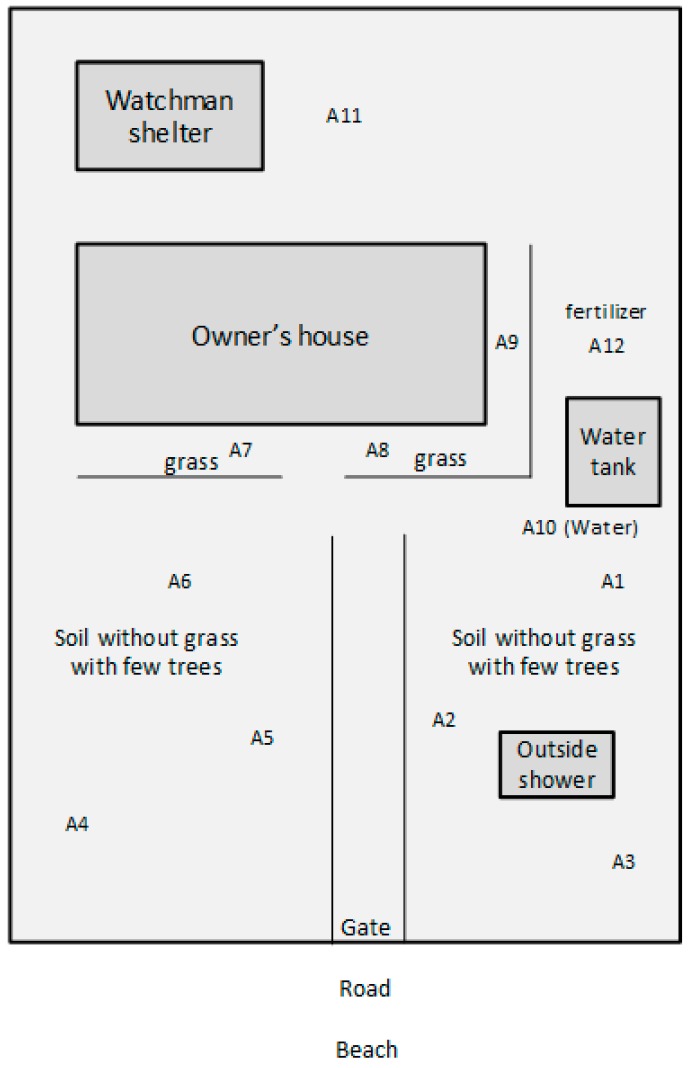
Schematic representation of the estate and yard of the fifth case from Mahajanga, Madagascar (#10). The estate is located along the seashore (separated by an asphalted road).

A soil investigation was carried out in the yard of the fifth case from Madagascar (#10). His house in Mahajanga was located on the seashore (separated by an asphalted road). Mahajanga (sometimes spelled Majunga) has a tropical wet and dry/savanna climate (Köppen-Geiger classification [13], with a pronounced dry season in the low-sun months, no cold season, and a wet season in the high-sun months (from November to April) (http://www.mahajanga.climatemps.com/). Sampling and culture of *B. pseudomallei* isolates from soil was done according to Appendix B. In brief, a total of 11 soil and water samples (Figure A1) were collected, among which was A12, a dark, dry, turf-like soil sold as fertilizer/manure by a local supplier. Sandy soil samples A4, A5, and A7-9 had been augmented with the dark soil A12.

The soil samples were processed as described in Appendix B, and subsequent cultures were spread on Ashdown’s agar plates (supplemented with colistin 50 mg/L) which were incubated at 37 °C. The plates were examined on a daily basis. Colonies with morphology typical for *B. pseudomallei* (pink/purple, flat, slightly dry, wrinkled-smooth but with surface roughness in the outer half of the colonies) were subcultured onto chocolate agar plates. Only samples A4, A8, and A12 gave rise to *B. pseudomallei*-like colonies on Ashdown medium. Identification of those isolates was confirmed by MALDI-TOF mass spectrometry (Bruker Biotyper, Bruker Daltonics, Germany) using the security-relevant database (*B. pseudomallei* with scores >2) and by PCR amplification targeting a genetic marker of the type III secretion system1 (TTS-1; 115-base-pair region within *orf2*) [14]. The three isolates were tested by disk diffusion on agar, and were found to be susceptible in vitro to amoxicillin/clavulanic acid, ceftazidime, imipenem, trimethoprim/sulfamethoxazole, levofloxacin, chloramphenicol, minocycline, but resistant to meropenem, according to the recommendations of the Antibiogram Committee of the French Society of Microbiology (CA-SFM guidelines 2015 for *Burkholderia cepacia*).

Total DNA was also extracted from 20 g of soil as described in Appendix B. As with the culture results, only soil samples A4, A8, and A12 were positive by PCR amplification for *B. pseudomallei*. MLST of soil isolates identified two novel STs: ST-1430 (4, 2, 3, 1, 5, 2, 1) and ST-1431 (1, 2, 3, 1, 5, 1, 1) from A12 and A4, respectively (Table 2). The isolate from sample A8 is still under investigation. The isolate from case #10 (the owner of the yard where the *B. pseudomallei* isolates were found) was also novel, being ST-1043 (4, 1, 3, 2, 5, 2, 1), which shares five alleles with soil isolate A12, but only two with soil isolate A4.

A consecutive investigation to trace back the origin of the fertilizer identified its origins as the surrounds of the Amborovy airport (15°40′56.59′′ S, 46°22′20.50′′ E); however, none of four soil samples taken from this location were positive for *B. pseudomallei*, either by culture or PCR.

Further genotyping (or WGS) of *B. pseudomallei* strains from the western Indian Ocean island region will play an important role in unraveling historical and contemporary epidemiological investigations in this region. Of historical importance are the postulated links between animal cases in Paris and Réunion Island and the presence of *B. pseudomallei* in the Antananarivo zoo in the 1970s [6,8], which are thought to illustrate the potential for *B. pseudomallei* to be disseminated across large geographic distances via infected animals or manure. It was previously reported that *B. pseudomallei* was transported from France to Réunion Island in the 1970s via an infected horse, which became ill with melioidosis after its arrival [6]. Although it has long been assumed that this horse contracted melioidosis in France, molecular characterization of this isolate would help to establish whether the horse in fact acquired *B. pseudomallei* from the environment in the Indian Ocean. In another instance, MLST characterization of *B. pseudomallei* isolates has enabled us to identify ST-27 isolates from both the Paris zoo outbreak and a 1977 soil isolate obtained from a zoo in Antananarivo. This previously unreported ST overlap suggests the possibility of transmission of *B. pseudomallei* between these two regions. However, it is not known whether ST-27 was introduced into France via Madagascar, or vice versa. Another possibility that should be considered is that of laboratory cross-contamination. On a contemporary level, molecular fingerprinting of *B. pseudomallei* from the western Indian Ocean island region will enhance surveillance measures, public health, and veterinary awareness of the disease, and epidemiological investigation to identify *B. pseudomallei* ‘hotspots’.

### 2.3. Mauritius

Case #4. The first recorded case of melioidosis in Mauritius occurred in January 2004 [15] when a 40-year-old woman on immunosuppressive treatment for systemic lupus erythematosus (SLE) was admitted to hospital with fever, nausea, and vomiting. She was initially started on ciprofloxacin, and was changed to cefotaxime and metronidazole after 48 h because of persistent fever. However, she developed cellulitis of her leg, became increasingly drowsy and confused, and passed away a week later. Her blood cultures became positive after 5 days of incubation, and by the time the organism was identified as *B. pseudomallei* by API 20NE and consistent with phenotypic characteristics, the patient had died. She lived in a low-socioeconomic suburb of Port Louis, and had never travelled abroad. According to her mother, her residence became very muddy at times of heavy rainfall and, although January is generally a wet month in Mauritius, January 2004 was wetter than average. The clinical isolate was subsequently referred to US Centers for Disease Control and Prevention for MLST genotyping, where it was found to be a novel ST, ST-1549 (alleles: 4, 12, 34, 2, 5, 2, 1).

Case #6. In 2006, *B. pseudomallei* was isolated from a swab from the leg wound of a Bangladeshi worker, who had been in Mauritius for less than one year and was admitted for a non-healing wound. The laboratory was prompted into speciating the organism by API 20E because of its positive oxidase test, colistin and aminoglycoside resistance, and the appearance of wrinkled colonies after 48 h. The patient was not diabetic and not generally unwell. He was treated with intravenous meropenem and oral trimethoprim/sulfamethoxazole. He was lost for follow-up, possibly because he returned to Bangladesh. It was difficult to obtain a clear history from the patient because of language difficulties, and although he stated that he had sustained the wound when he had fallen off his bed in Mauritius, it is possible that he acquired the infection in Bangladesh, with reactivation of infection from a latent focus occurring several months later. Since then, there have been no further documented cases of melioidosis in Mauritius. 

### 2.4. Réunion Island 

Case #7. In addition to the three imported cases from Madagascar described hereinabove (cases #1, #5 and #13), an autochthonous case was hospitalized in Réunion Island in 2012 [16]. A 57-year-old patient presenting with type 2 diabetes mellitus and hypertension was admitted to Saint-Pierre Hospital with fever (39 °C), an elevated C-reactive protein (248 mg/L), and acute urinary retention requiring the placement of a urinary catheter. Empirical ceftriaxone treatment was initiated, based on an early diagnosis of acute bacterial prostatitis (tender and enlarged prostate without any other physical abnormalities). The urine and three blood cultures were positive for *B. pseudomallei* using the Vitek Compact (bioMérieux, La Balme-Les-Grottes, France). The bacterium was reported as being sensitive to ceftazidime, imipenem, doxycycline, and resistant to all aminoglycosides and colistin according to EUCAST criteria [18]. The therapy was switched to iv ceftazidime and doxycycline. When he recovered he was discharged, but used a peripheral catheter to complete a 4-week course of ceftazidime, and oral doxycycline and trimethoprim/sulfamethoxazole were given to complete 5 months of eradication treatment. The identification of *B. pseudomallei* was confirmed by MALDI-TOF mass spectrometer (MS) (Bruker Biotyper, Bruker Daltonics, Bremen, Germany), PCR targeting type III secretion system genes, and 16S rDNA sequencing. This patient had always lived in Réunion Island, except for three trips of one week to Mauritius five years before becoming ill. No risk factors for exposure were identified.

Case #14. The last reported case (2017) was an imported case in a 40-year-old man who lived in Indonesia, and was working as a fitter’s mate on a cruise ship, which stopped in southeast China and Singapore before cruising to Réunion Island [2]. He had no significant past medical history but developed a fever 15 days after departure from Singapore, and was treated with amoxicillin/clavulanic acid. Five days later, when his condition worsened, he was transported by helicopter from the ship to Saint Denis hospital, Réunion Island. He was admitted with fever (40.6 °C), extensive skin pustules on the face, hepatomegaly, septic shock, and multiple organ failure, requiring intubation. TB-CT scan revealed cerebral venous thrombosis, multiple bilateral lung abscesses, a liver abscess, splenomegaly, and hepatomegaly. Despite immediate antimicrobial therapy with high doses of meropenem (continuous treatment 6 g/day) and amikacin, blood cultures remained positive until day 10. A second TB-CT scan and magnetic resonance imaging of the brain were performed, which showed two subdural empyemas (of 9.5 mm). Antibiotic treatment was changed to ceftazidime (12 g/day by continuous infusion) and the patient’s condition improved on day 15, and he was discharged from the ICU on day 70. The last imaging (day 63) showed a reduction in the size of the bilateral lung abscesses, an almost complete regression of the splenic and hepatic abscesses, and a significant reduction of the cerebral abscesses. Blood cultures, respiratory samples and skin abscesses were positive for a *Burkholderia* species identified as *B. thailandensis* by MALDI-TOF MS but confirmed as *B. pseudomallei* by qPCR targeting three genetic markers of the type III secretion system (*orf1*, *orf13*, and *BsSCU2*), as previously reported (see case #13 from Madagascar). The strain was susceptible in vitro to amoxicillin/clavulanic acid (2/1) (MIC of 2 mg/L), ceftazidime (MIC of 1 mg/L), meropenem (MIC of 0.75 mg/L), imipenem (MIC of 0.38 mg/L), trimethoprim/sulfamethoxazole (1/19) (MIC of 0.064 mg/L), and doxycycline (MIC of 0.25 mg/L).

### 2.5. Seychelles 

In January 2013, two patients were hospitalized at Seychelles Hospital with a history of fever, cough, shortness of breath, and chest pain. Their condition deteriorated after admission and they were transferred to the ICU [17]. They were both cooks from the same facility and both had a history of illicit substance abuse. The Seychellois, who had no history of travel, passed away, whereas the other cook, who survived, was from Mauritius, but had been in the Seychelles for the previous ten years. The initial diagnosis was MERS-CoV, legionellosis, or possible tuberculosis. Lung and blood samples were sent to the Health Protection Agency in England and to the National Institute for Communicable Diseases in South Africa for investigation. Samples from both patients grew *B. pseudomallei*, confirmed by qPCR and serology (high IgG titers consistent with a recent infection). The bacterial isolates were sensitive to a wide range of antibiotics, and one of the isolates had a novel ST, ST-1432 (4, 2, 3, 2, 5, 2, 1). A third possible case, who worked at the same premises but not in the kitchen, was identified through active case finding. This third patient originally came from Madagascar. He had equivocal antibody results (1:4000) suggestive of possible exposure to *B. pseudomallei*. The possibility of a point source of contamination was investigated, but no common source of infection was identified.

### 2.6. Genetic and Genomic Relatedness between Strains Isolated in the Western Indian Ocean

In recent years, whole-genome sequencing (WGS) has become a cost-effective method for strain genotyping, including for MLST. Using WGS data for phylogenomic analyses has also been instrumental in unravelling the early origins of *B. pseudomallei* in Australia, and its subsequent dissemination to tropical regions across the globe, including the western Indian Ocean region. Three studies to date have used WGS to investigate *B. pseudomallei* strains from western Indian Ocean islands [19,20,21]. Sarovich and colleagues [19] were the first to identify an Asian origin for the Madagascan *B. pseudomallei* strains included in the phylogenetic analysis. Their study showed that the introduction of this bacterium into Madagascar was likely associated with the migration of Austronesian peoples (or their animals) from Indonesian Borneo approximately 2000 years ago [19]. *B. pseudomallei* was subsequently transmitted from the western Indian Ocean region to mainland Africa, and then the Americas, during the Atlantic slave trade [19,20]. The mechanism/s of *B. pseudomallei* dissemination to other western Indian Ocean islands is not yet well understood, but it is likely that it has also been anthropogenically driven. It is also unknown how many of the Indian Ocean islands harbor *B. pseudomallei*, with confirmed cases found only in the most populous islands (Madagascar, Mauritius, Réunion Island, and Seychelles). 

Table 2 presents the ST and alleles for 8/13 cases identified in the western Indian Ocean, as well as for the two soil isolates from the garden of case#10 from Mahajanga, Madagascar. MLST analyses have shown that there is no ST overlap between islands, and the STs identified on these islands are not found elsewhere, with the exception of ST-1053, which has been located in both Madagascar [19] and in an imported American case thought to have originated in Ghana [21], although the possibility that this patient had travelled to Madagascar cannot be ruled out. It is also important to stress that the ST of case #13 (hospitalized in Saint Denis, La Réunion, but originally from Mahajanga, Madagascar) and E1 (soil from Mahajanga, Madagascar) are identical (ST-1430), indicating the possibility that the Mahajanga environment was the source of infection in case #13.

## 3. Current Recommendations and Availability of Measures against Melioidosis

So far, only sporadic melioidosis has been reported in this region, but the increasing number of reported cases ([2], this study) should raise awareness among all those working in the healthcare sector and public health policy makers of the possibility of melioidosis, especially during the rainy season. Doctors and laboratory staff should know how to confirm the diagnosis and treat the disease. Indeed, the association between rainfall and melioidosis has been well demonstrated in hyperendemic regions, with 75% and 85% of cases occurring during the wet season in northeastern Thailand and northern Australia [22,23]. The contaminated soil in the garden of case #10 highlights the risk of occupational or recreational exposure to *B. pseudomallei*, and predisposing risk factors, such as diabetes mellitus, chronic lung or renal diseases, hazardous alcohol use, or thalassaemia, should be considered when gardening or cultivating in endemic zones. Additionally, *B. pseudomallei* has been reported to infect a wide range of animals, such as horses, sheep, goats, cattle, pigs or cats, with anecdotal reports of transmission from animals to humans [22]. Contaminated secretions and excreta represent a possible source of exposure or environmental contamination. As the bacterium typically enters the body through pre-existing cutaneous lesions, including minor trauma such as insect bites, and develops in the wound, contact with contaminated waste may represent an underestimated source of infection. 

As with many other tropical regions, melioidosis is probably considerably underreported in the western Indian Ocean islands, and fostering its reporting within national surveillance systems would help to improve monitoring of the incidence of this disease in the western Indian Ocean. Soil (including domestic yards and gardens [24]), surface water and animal waste sampling to test for the presence of *B. pseudomallei* could also be proposed, especially in and around the city of Mahajanga, which has been associated with all the cases from Madagascar. In addition, a seroprevalence survey could be undertaken among all farmers and gardeners in this area.

## 4. Awareness of Melioidosis

To improve prevention and control of melioidosis, we recommend that physicians consider melioidosis in the differential diagnosis of patients with (1) acute febrile illnesses; (2) risk factors for melioidosis [22] (impaired neutrophil function, diabetes mellitus, pre-existing renal or lung diseases, malignancy, thalassemia and/or excessive alcohol consumption); and (3) compatible occupational or recreational exposure history (soil and water exposure: i.e., farmers, gardeners). However, healthy people can also get the disease if they are infected with a high bacterial load, such as contact with muddy soil without good hand and foot protection. Clinical manifestations are broad, and range from subclinical infection to localized abscess formation, pneumonia, and systemic sepsis. Pneumonia is the most commonly recognized presentation of melioidosis associated with high fever, significant muscle aches, and chest pain [25]. Melioidosis could easily be confused with tuberculosis or even plague, particularly in Madagascar, where this disease is prevalent, or with pneumonia caused by other pathogens. Acute melioidosis septicemia is the most severe form of the infection. It presents as a typical sepsis syndrome with hypotension, high cardiac output, and low systemic vascular resistance. In many cases, a primary focus in the soft tissues or lungs can be found. The syndrome, usually in patients with underlying risk factors, is characteristically associated with multiple abscesses involving soft tissues, the lung, the liver, and spleen. Blood culture is an effective way to diagnose many melioidosis cases, as 50% or more of infections have bacteremia [26].

With the exception of a localized hospital-based study of patients with hyperthermia (≥39 °C), with or without shivering and septic shock, in Mahajanga in 2012, which led to the recognition of cases #8 and #9, no systematic case finding of *B. pseudomallei* infection has been conducted in the western Indian Ocean islands. Generally, there is no awareness of melioidosis in the population and among clinicians, because its diagnosis has been rare, despite the very high prevalence of diabetes in Mauritius. Some laboratory technicians and the clinical microbiologists who diagnosed the cases described above are aware that *B. pseudomallei* is a possibility when they isolate an oxidase-positive Gram-negative bacillus that is resistant to colistin and aminoglycosides, and which produces wrinkled colonies and/or has an earthy smell. However, this knowledge is not universal.

## 5. Current and Future Challenges

Further studies are needed in order to investigate the true burden of melioidosis in the western Indian Ocean, and especially Madagascar, since it is likely to be higher than currently reported. This could be done by enhancing diagnostic microbiology provision and surveillance systems, training and education of healthcare staff, serological studies and environmental investigations.

## Figures and Tables

**Table 1:** Case descriptions of patients who acquired melioidosis after visiting Mahajanga, Madagascar.

Year	2004	2005	2012	2013	2013	2016
Case	#1	#5	#8	#9	#10	#13
Reference	[9]	[10]	[11]	[11]	[12]	[2]
Sex	Male	Male	Male	Male	Male	Male
Age	60	58	52	45	44	63
Strain ID (sequence type) *	4419 (ST-1260)	4420 (ST-1433)	3240 (ST-1053)	3241 (ST-1054)	4416 (ST-1043)	2017-012 (ST1430)
Allele profile	1, 12, 34, 2, 5, 2, 1	4, 1, 34, 2, 5, 2, 1	4, 12, 3, 2, 5, 2, 1	4, 12, 34, 1, 5, 2, 1	4, 1, 3, 2, 5, 2, 1	4, 2 ,3, 1, 5 ,2, 1
Occupation	Not known	Retired	Rural rice farmer	Rice, sugar cane, and tobacco farmer	Gardener	Not known
Risk factors	Heavy drinker (1 L/day) and smoker (20 cigarettes/day); cachexia	Smoker (35 cigarette packs/year); unremarkable medical history	Diabetes	Diabetes. A history of furunculosis for several months	No diabetes mellitus	
1st admission	CHD Félix Guyon, Saint Denis, Réunion Island (24.05.2004)	Hospital in Antananarivo (March 2005)	Androva University Hospital in Mahajanga (July 2012)	Androva University Hospital in Mahajanga (May 2013)	CHU-AP, Mons, Belgium (16.03.2013)	CHD Félix Guyon, Saint Denis, Réunion Island
2nd admission		Groupe Hospitalier Sud Réunion, Saint Pierre, Réunion Island			CHU-AP, Mons, Belgium (April 2013) due to identification of the causative agent and symptoms	
Previous history	Lived in Madagascar (Mahajanga); lived for an unknown period of time in Vietnam 20 or 30 years before the onset of the disease	Spent most of his life in France but lived for the past 5 years in Madagascar before hospitalisation in Antananarivo. He had also travelled for short periods in Tunisia, Turkey and Mauritius. The first symptoms appeared during the last days of a stay (couple of weeks) in Mahajanga			Frequent travels to Mahajanga for entertainment (beach sports and fishing) lasting usually for 3 weeks every 3 or 4 months including during the rainy season	Had consulted the Hospital in Mahajanga 4 days before being admitted to CHD Félix Guyon, Saint Denis
Outcome	Discharged	Clinical improvement and discharged	Died 3 days later	Died 2 weeks after admission and two days after ceftazidime treatment	Discharged a few days after his clinical status remained stable, with decreasing inflammatory syndrome and fever.	Discharged a few days after his clinical status remained stable, with decreasing inflammatory syndrome and fever.	Died on day 1 after his admission

* According to the *B. pseudomallei* multilocus sequencing typing (MLST) database (https://pubmlst.org/bpseudomallei/).

**Table 2:** Genetic typing of *B. pseudomallei* isolated from patients in the western Indian Ocean, and from the soil from the yard of case 9, Mahajanga, Madagascar.

Case [Ref.]	Year	Place of Diagnosis	City (Country) Visited within 12 Months of Diagnosis	Allele Profile								MLST Type	Remark
#1 [9]	2004	Réunion Island	Mahajanga (Madagascar)	1	12		34	2	5	2	1	1260	
#2 [9]	2004	Réunion Island *	0										Not typed, nosocomial cases
#3 [9]	2004	Réunion Island *	0										Not typed, nosocomial cases
#4 [15]	2004	Mauritius	0	4	12		34	2	5	2	1	1549	ST1549 is a single locus variant of ST1053 (case #8), ST1054 (case #9), ST1260 (case #1), and 1433 (case #5)
#5 [10]	2005	Madagascar (1st admission)/Réunion Island (2nd admission)	Antananarivo and Mahajanga (Madagascar)	4	1		34	2	5	2	1	1433	ST1433 is a single locus variant of ST1549 (case #4)
#6 [This study]	2006	Mauritius	Bangladeshi worker										Not typed
#7 [16]	2012	Réunion Island	None										Not typed
#8 [11]	2012	Madagascar	Mahajanga (Madagascar)	4	12		3	2	5	2	1	1053	ST1053 is a single locus variant of ST1043 (case #10), ST1432 (case #11), ST1549 (case #4)
#9 [11]	2013	Madagascar	Mahajanga (Madagascar)	4	12		34	1	5	2	1	1054	ST1054 is a single locus variant of ST1433 (case #5), ST1549 (case #4)
#10 [12]	2013	Belgium	Mahajanga (Madagascar)	4	1		3	2	5	2	1	1043	ST1043 is a single locus variant of ST1053 (case #8), ST1432 (case #11), ST1433 (case #5)
#11 [17]	2013	Seychelles **	Unknown	4	2		3	2	5	2	1	1432	ST1432 is a single locus variant of ST1043 (case #10), ST1430 (soil E1)
#12 [17]	2013	Seychelles **	Unknown										Not typed
#13 [2]	2016	Réunion Island	Mahajanga (Madagascar)	4	2		3	1	5	2	1	1430	Same ST than E1. ST1430 is a single locus variant of ST1432 (case #11).
#14 [2]	2017	Réunion Island	Southeast Asia										Not typed
Environmental isolates													
E1 [This study]	2014	Soil from the garden of case 9 (A4)	N/A	4	2		3	1	5	2	1	1430	Same ST as case #13. ST1430 is a single locus variant of ST1432 (case #11).
E2 [This study]	2014	Soil from the garden of case 9 (A8)											Typing in progress
E3 [This study]	2014	Soil from the garden of case 9 (A12)	N/A	1	2		3	1	5	1	1	1431	

* Cases #2 and #3 were two nosocomial cases acquired from an endoscope used for Case 1. ** Cases #11 and #12 were two cooks from the same facility, hospitalized at the same time.

## Appendix B. Materials and Methods

### Appendix B.1. Soil Sampling and Culture of B. pseudomallei from Soil and Water

Eleven soil samples and one artesian well water sample were taken from the yard of case #10—(Mahajanga, Madagascar) in 2014 (see Figure A1, Appendix A), one year after the owner fell sick.

*Soils*: Twenty grams of each soil sample, identified as A1 to A10 and A12, were shaken at 220 rpm at 37 °C for 24 h in 20 mL of distilled water. Ten ml of supernatant were removed and were placed into 30 mL of modified Ashdown’s broth (+colistin 50 mg/L). After 48 h, 10 μL of supernatant were spread on modified Ashdown’s medium (+gentamicin at 8 mg/L). The plates were examined on a daily basis. Colonies with morphology typical for *B. pseudomallei* were subcultured onto chocolate agar.

*B. pseudomallei* colonies were identified by basic screening tests (Gram stain, oxidase test), rapid detection of the capsular polysaccharide (CPS) produced by *B. pseudomallei* (Active Melioidosis Detect^TM^ (AMD) rapid test (*InBios*)) [27], MALDI-TOF mass spectrometry, and molecular identification based on the amplification of the *B. pseudomallei* specific target TTS1 (see below).

*Water:* Sample A10 comprised 1 liter of water from the artesian well. The water was filtered through a membrane (pore size 0.45 µm, diameter 47 mm) on a filtration ramp (Combisart Multi-Branch Systems Manifolds, Sartorius, Göttingen, Germany). The membrane was subsequently placed in Ashdown broth (supplemented with colistin 50 mg/L). After incubation for 48 h, the membrane was transferred onto an Ashdown agar plate (containing gentamicin at 8 mg/L) and incubated for 48 h. No suspicious colonies of *B. pseudomallei* were detected.

### Appendix B.2. Molecular Detection of B. pseudomallei from Soil

DNA was extracted from 20 g of soil as previously described [28]: In brief, 20 g of soil was incubated for 48 h with shaking at 37 °C in 20 mL of selective modified Ashdown’s broth [29] containing colistin; the sample was then centrifuged twice, and the pellet processed for DNA extraction using a modified protocol of the PowerSoil DNA isolation kit (MoBio Laboratories, Carlsbad, NM, USA). Modifications included the addition of 0.8 mg of aurintricarboxylic acid (ATA) and 20 μL of proteinase K (20 mg/mL). DNA was eluted in 100 μL of 10mM Tris HCl. *B. pseudomallei* DNA was detected by conventional PCR targeting a 115 bp stretch of the *B. pseudomallei* specific *orf2* of type III secretion system (TTS1) as described below. Briefly, 4 μL of DNA were amplified in duplicates in 25 μL volumes.

### Appendix B.3. Identification of B. pseudomallei Isolates by PCR

The PCR was conducted in a final reaction volume of 25 µL containing 2 µL of DNA, 0.5 U of FIREPol DNA polymerase, 1× FIREPol Master Mix Ready to Load (2.5 mM MgCl_2_), 100 µM of dNTP (Solis Biodine, Tartu, Estonia), and 200 nM of each primer. Primers used to amplify a 548 bp of the *B. pseudomallei* TTS1 gene were BPTT4176F (CGTCTCTATACTGTCGAGCAATCG) and BPTT4290R (CGTGCACACCGGTCAGTATC) [14]. The PCR was performed on a thermocycler (Multigene, Labnet, Woodbridge, NJ, USA) with an initial denaturing step at 95 °C for 1 min, an extension step at 95 °C for 60 s, 54 °C for 60 s, and 72 °C for 2 min for 30 cycles; and a final extension step at 72 °C for 10 min. Samples were separated by agarose gel electrophoresis, stained with ethidium bromide, and visualized with the gelscan (Infinity, VX2-1126MX, Montreal Biotech, Dorval, QC, Canada).

### Appendix B.4. MLSTF

The molecular typing tool multilocus sequence typing (MLST), based on sequence polymorphisms in seven genetically stable housekeeping genes, was developed by Brian Spratt at Imperial College London as the initial phylogenetic tool for population analysis of *B. pseudomallei* [7].

PCR conditions were as follows: for each locus, the PCR mixture contained 0.5 U FIREPol DNA polymerase (Solis Biodine, Tartu, Estonia), 200 nM of primers, 1× buffer master mix FIREPol Master Mix Ready to Load (2.5 mM MgCl_2_), 100 µM of dNTP, and 2 µL of extracted DNA in reaction volumes 25 µL. These were carried out in a 96-well plate format, with initial denaturation at 95 °C for 4 min, followed by 30 cycles of 95 °C for 30s, 62 °C for 30 s, and 72 °C for 60 s. The samples were maintained at 72 °C for a further 10 min, cooled to 4 °C, and stored at −20 °C. The DNA fragments were sent for sequencing (each strand; forward and reverse) to Beckman Coulter with the primers used in the initial PCR amplification.

For each locus, sequences obtained were analyzed using the MLST database containing the sequences of alleles, allelic profiles, and information about the *Burkholderia* isolates, with analysis tools. This tool is stored on a publicly-available web-based database, allowing comparisons to be made between isolates from different laboratories (https://pubmlst.org/bpseudomallei/). MLST is the gold standard for comparison of isolates from different geographical locations due to high inter-laboratory reproducibility.
